# Correlation of Cell Proliferation with Surface Properties of Polymer-like Carbon Films of Different Thicknesses Prepared by a Radio-Frequency Plasma CVD Process

**DOI:** 10.3390/ma15134466

**Published:** 2022-06-24

**Authors:** Kazuya Kanasugi, Hiroaki Eguchi, Yasuharu Ohgoe, Yoshinobu Manome, Ali Alanazi, Kenji Hirakuri

**Affiliations:** 1 Department of Electrical and Electronic Engineering, Faculty of Engineering, Tokyo Denki University, 5 Senju Asahi-cho, Adachi-ku, Tokyo 120-8551, Japan; 21kmj12@ms.dendai.ac.jp (H.E.); hirakuri@mail.dendai.ac.jp (K.H.); 2 Division of Electronic Engineering, Faculty of Science and Engineering, Tokyo Denki University, Ishizaka Hatoyama, Saitama 350-0394, Japan; yasuharu@mail.dendai.ac.jp; 3 Core Research Facilities, The Jikei University School of Medicine, 3-25-8, Nishi-shinbashi, Minato-ku, Tokyo 105-8461, Japan; manome@jikei.ac.jp; 4 Applied Medical Sciences College, King Saud University, Riyadh 11451, Saudi Arabia; asanazi@ksu.edu.sa

**Keywords:** cell proliferation, polymer-like carbon films, optical classification, film thickness

## Abstract

In this study, correlation of cell proliferation with surface properties of the polymer-like carbon (PLC) films of different thicknesses prepared by radio-frequency plasma CVD are investigated. Four PLC samples were prepared via radio frequency plasma chemical vapor deposition on Si substrates. Each PLC film was analyzed using spectroscopic ellipsometry to determine its thickness, refractive index (n), and extinction coefficient (k); the thickness ranged from 29.0 to 356.5 nm. Based on their n–k plots, all the samples were classified as PLC-type films. The biological response of the PLC films was evaluated in vitro using a cell culture. The samples with relatively thick PLC films (>300 nm) exhibited stronger cell proliferation properties than those with thinner films. Moreover, the results of the surface analysis showed no significant differences in the surface composition of those PLC samples, as analyzed using X-ray photoelectron spectroscopy, but that as the PLC films became thicker, their surfaces became rougher on the nanoscale and their wettability improved. Overall, this study showed that careful control of the film growth of PLC films, which affects their surface properties, is essential for their use in bio-interface applications.

## 1. Introduction

Diamond-like carbon (DLC) films are disordered, thin carbon films containing sp^2^ hybrid orbital bonds, sp^3^ hybrid orbital bonds, and hydrogen bonds. The molecular structure of DLC films is highly dependent on the deposition conditions, such as the deposition method and precursors, and a wide range of sp^2^/sp^3^ ratios and hydrogen contents can be achieved by controlling these factors [1]. These films are also known to exhibit attractive characteristics, such as high hardness, corrosion resistance, and gas barrier properties, depending on the DLC film structure [2,3,4]. In addition, DLC films possess a high degree of biological affinity, as indicated by their antithrombotic and cytophilic properties. Thus, they have potential applicability in various biological interface applications, such as artificial hip joints and arterial stents [5,6]. However, for an average user of DLC coatings, it can be challenging to select the appropriate film deposition method and conditions required to achieve the desired DLC film structure and properties.

DLC film deposition methods can be broadly divided into two major categories: physical vapor deposition (PVD), which uses solid graphite as a precursor, and chemical vapor deposition (CVD), which uses hydrocarbon gas. DLC films formed using PVD exhibit relatively low hydrogen content and properties such as abrasion, scratch, and corrosion resistance [1]. Conversely, DLC films formed using CVD exhibit relatively high hydrogen content and superior flexibility [1]. “DLC films” is a general term for thin, disordered carbon films, and there are a variety of different DLC films with varying characteristics. The molecular structure of these DLC films has been debated for a long time. The most popular model, proposed by Robertson, states that DLC films possess a three-dimensional structure based on the number of sp^2^/sp^3^ bonds and hydrogen content [1].

Recently, Hiratsuka et al. proposed an optical classification method based on the optical constants of individual DLC films [7]. This classification method uses the n–k plot (λ = 550 nm) based on the refractive index (n) and extinction coefficient (k), determined via spectroscopic ellipsometer (SE) analysis. DLC films can be categorized into six different types: amorphous carbon (a-C), hydrogenated amorphous carbon (a-C:H), tetrahedral amorphous carbon (ta-C), hydrogenated tetrahedral amorphous carbon (ta-C:H), polymer-like carbon (PLC), and graphite-like carbon (GLC) [7].

In previous studies, we investigated the relationship between the surface properties and the cell proliferation properties and optical constants of the four categories of DLC (i.e., a-C, a-C:H, PLC, and GLC) under different deposition methods and conditions. The results of these investigations confirmed that the a-C and GLC types with relatively high extinction coefficients had larger C=O bond ratios and stronger cell proliferation-promoting properties than the a-C:H and PLC types, which had relatively low extinction coefficients [8]. This is thought to be due to the fact that the zeta potential and hydrophilicity improve with increasing C=O bond ratios on the DLC surface. In addition to surface composition, the micro- and nanostructure of cell scaffold materials have previously been reported to strongly influence the behavior of cells in terms of activation, adhesion, and proliferation [9,10]. 

Harigai et al. have shown that the surface roughness of DLC films deposited on Si substrates by radio-frequency plasma CVD increases on a nanoscale in proportion to the film thickness (deposition time) [11]. However, the effect of the change in film thickness on the relationship between the surface properties and cell proliferation has not been extensively investigated in previous studies.

In this study, among the many DLC types, the PLC type was focused on; four PLC samples with different film thicknesses were prepared on Si substrates by controlling the deposition time of the high-frequency plasma CVD process. The relationship between the surface properties with the growth (thicknesses) of the PLC films and cell proliferation was then investigated.

## 2. Materials and Methods

### 2.1. PLC Film Deposition Conditions

A parallel plate with a 13.56 MHz, radio-frequency plasma CVD device (RF-PECVD; PED-401, ANELVA, Ltd., Kanagawa, Japan) was used to deposit a PLC film onto an Si {100} substrate (10 × 10 mm) placed on the cathode. The samples were prepared for cell culture and analysis, respectively. This RF-PECVD method is one of the most common PLC film generation methods used in industrial settings [12]. Prior to PLC deposition, the vacuum chamber was evacuated to 1 Pa. The PLC film deposition conditions are presented in Table 1. In this study, the PLC film thickness of each sample was modified and controlled by varying the film deposition times from 0.8 to 13.2 min. In addition, ultrasonic cleaning using acetone and ethanol was performed for 10 min to remove pollutants from the surface of the Si substrate prior to the formation of each film. Furthermore, the natural oxide film present on the surfaces of the Si substrates was not intentionally removed before deposition. 

### 2.2. Spectroscopic Ellipsometer Analysis

To determine the film thickness and optical constants of each PLC film sample, a phase-modulated spectroscopic ellipsometer (SE; UVISEL PIUS, HORIBA, Ltd., Kyoto, Japan) was used. In this SE analysis, the angle of incidence was fixed at 70°, and the reflection amplitude ratio angle (ψ) and phase difference (Δ) were measured for s-polarized and p-polarized light with wavelengths ranging from 191 to 2066 nm (photon energy: 0.6 to 6.5 eV). The measurement interval was 0.1 eV with an elliptical spot of size 1 × 3 mm. The film thickness was measured with a resolution of 1 A. In addition, regression analysis was performed based on the SE spectrum by using a virtual thin-film model that assumed a PLC film layer to substrate ratio with a small chi-square (χ^2^) value in order to determine the refractive index (n) and extinction coefficient (k). Furthermore, the Tauc–Lorentz model was used for regression analysis of the PLC film layer [7]. The resolutions of the refractive index and extinction coefficient were 0.001.

### 2.3. Structure Analysis

The structure of the PLC films was evaluated using Raman spectroscopy (SpectraPro 2750, Princeton Instruments, Acton, MA, USA). In addition, the samples for analysis were not exposed to ultraviolet sterilization. For Raman analysis, the laser power was set to 1 mW, the laser wavelength to 532 nm, and the exposure time to 30 s. It is well known that the Raman spectrum of PLC films has a D-peak (at approximately 1350 cm^−1^) related to the disordered structure and a G-peak (at approximately 1550 cm^−1^) related to the graphitic structure [1]. In this experiment, these peaks were separated using a Gaussian function and used to calculate the I_D_/I_G_ intensity ratio.

### 2.4. Cell Culture Testing

The cell proliferation properties of the PLC films were evaluated in vitro using a cell culture test with mouse-derived fibroblasts (NIH-3T3) and osteoblasts (MC-3T3). These cells are standard model cells that are widely used to evaluate the biological response of DLC films [5,13,14]. As a control, Si substrates without PLC films deposited were also evaluated. This Si substrate has been previously reported to have good cell affinity [15]. Before cell culturing, all PLC samples were sterilized for 1 h using 253.7 nm ultraviolet light. Thereafter, each of the PLC samples was placed on a 12-well cell culture plate, and each cell was cultured on the PLC film surface for 72 h (n = 3). The culture conditions for each cell are listed in Table 2. 

After culturing, trypsin (0.25% *w*/*v* Trypsin-1 mmol/L EDTA-4Na Solution with Phenol Red, Wako Ltd., Osaka, Japan) was used to remove the surviving cells attached to the PLC surface. Thereafter, CellTiter-Blue^®^ Viability Assay (Promega, Madison, WI, USA) was added to each sample to evaluate the number of surviving cells. When this assay was added to the samples, the surviving cells converted the redox dye resazurin into fluorescent resorufin. The number of surviving cells was measured (n = 5) based on the fluorescence emission intensity (wavelength: 580 nm), which was determined using a plate reader (2300 En Spire, Perkin Elmer Ltd., Waltham, MA, USA). For the purposes of this experiment, cell proliferation was defined as the number of surviving cells after 72 h. Cell proliferation is expressed as mean ± standard deviation (SD). After PLC film lyophilization, the cell morphology on the PLC film was observed after 72 h using a scanning electron microscope (Regulus 8100; HITACHI, Ltd., Tokyo, Japan).

### 2.5. Statistical Analysis

The experiments for the cell culture evaluation were performed in triplicates. Subsequently, a multiple comparison test based on the Tukey method was conducted to check whether the differences among the samples were statistically significant [16]. In this test, a significance level of ≤5% was considered significant. Therefore, *p* < 0.05 indicates a significant difference in cell proliferation between the PLC samples with different film thicknesses.

### 2.6. Surface Analysis

For the surface analysis, the wettability, surface composition, and surface roughness of each PLC sample were evaluated. The wettability of the PLC films was evaluated based on contact angle measurements with pure water (2 μL) at room temperature (20 °C). The pure water used in this experiment was membrane-filtered deionized water purified by ion exchange. The contact angle was calculated using the θ/2 method [17]. Furthermore, in this contact angle tactile angle evaluation, the drop position of the liquid was changed, and the evaluation was repeated 10 times. The chemical composition of the PLC surface was analyzed using X-ray photoelectron spectroscopy (XPS; JPS-9000MC, JEOL, Ltd., Tokyo, Japan). Furthermore, In Furthermore, XPS non-monochromatic radiation (MgKα source, 10 mA, 10 kV) was used to measure the survey spectra and the C1s narrow peak. The photoelectron extraction angle was set to 45 degrees and the path energy to 10 eV. The analysis was conducted by standardizing the maximum peak value of C1s at 1. In addition, shift correction was performed to ensure that the maximum peak of C1s occurred at 284.5 eV. Furthermore, the C1s peak of each PLC sample obtained was waveform-separated into C-C sp^2^, C-C sp^3^, C-O, C=O and O=C-O. All binding energies were calculated with reference to the carbon 1s peak of the surface at the C-C sp^2^ bond (approximately 284.0 eV) [8,18]. In addition, Nitta et al. reported that the introduction of oxygen functional groups, such as C-O, C=O, and O=C-O, resulted in hydrophilicity and a negative charge [18]. The PLC film surface roughness was analyzed using an atomic force microscope (AFM; SPM-9700HT, SHIMADZU, Ltd., Kyoto, Japan). The root-mean-square roughness (RMS) was derived via AFM analysis in the contact mode over a range of 10 × 10 μm.

## 3. Results and Discussion

### 3.1. Classification of DLC Types Based on n–k Plots

**Figure 1:**
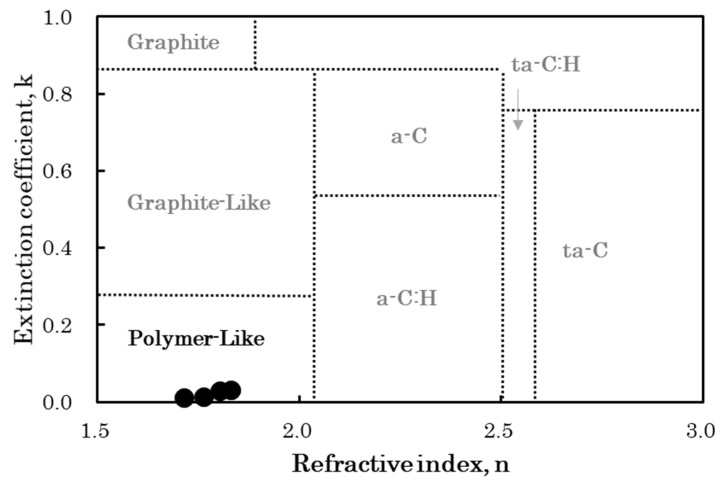
DLC film types based on n–k plots [7,19].

The film thickness, refractive index, and extinction coefficient of four types of PLC samples with different deposition times were evaluated using SE analysis. The SE analysis results are shown in Table 3, and the n–k plots of the samples are shown in Figure 1. The χ^2^ values were relatively small in the SE regression analysis, suggesting that the fitting was performed accurately. The thickness of the film is shown as the mean value ± the fitting error. The PLC film thickness increased, ranging from 29.0 to 356.5 nm, in proportion to the RF-PECVD deposition time. In addition, the optical constants of the PLC films varied according to the film thickness, with the refractive index and extinction coefficient values ranging from 1.714 to 1.830 and 0.012 to 0.031, respectively. Because the resolutions of the refractive index and extinction coefficient were 0.001, the optical constants between the samples were considered to be significantly different. However, based on their n–k plots, all four PLC samples were classified as PLC-type DLC films. This means that, at least in the film thickness range of this experiment, the optical constants did not change significantly enough to change the type of PLC. According to Hiratsuka et al., the refractive index is high when the proportion of C-C sp^3^ bonds is high and the hydrogen content is low [7]. Additionally, they reported that the smaller the number of π-electrons in the graphite structure, the lower the extinction coefficient [7]. Accordingly, it can be inferred that DLC samples classified as PLC are films with a relatively high hydrogen content and a small number of π–π * bonds. Therefore, the refractive index of DLC films including PLC-type is correlated with film hardness [7]. This suggests that the PLC-type films formulated in this experiment were softer than the other film types.

### 3.2. Structural Changes with Growth of the PLC Films

**Figure 2:**
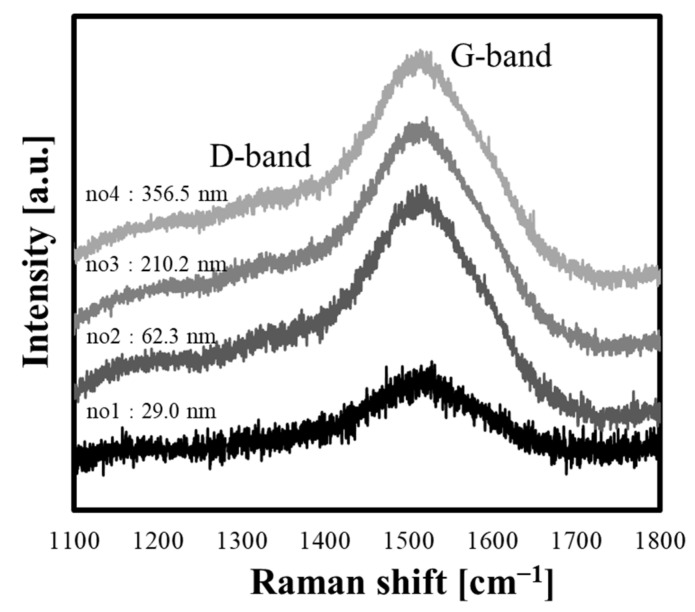
Raman spectra of the polymer-like carbon (PLC) films with different thicknesses.

**Figure 3:**
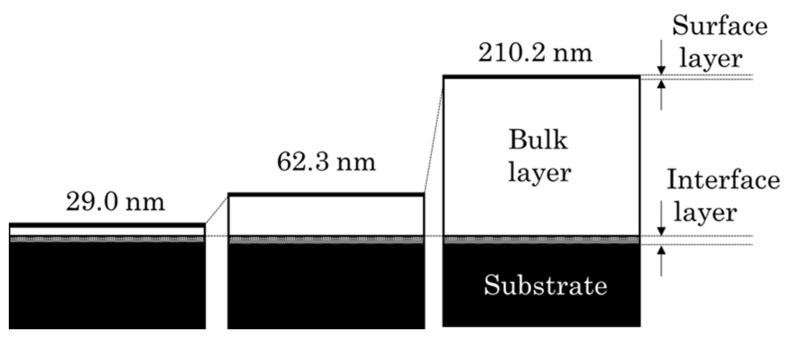
PLC film structural model prepared by a radio-frequency plasma CVD process.

The structure of the four types of PLC samples classified based on the n–k plots was evaluated via Raman spectroscopy. The Gaussian fitting results of the Raman spectra are shown in Table 4, and the Raman spectra of the samples are shown in Figure 2. All PLC samples prepared in this experiment had broad spectra with a G-peak and D-peak, which are characteristic of PLC. The position of the G-peak for the PLC film thickness was found to be slightly shifted toward the high wavenumber side in the range 1515.77–1524.85 cm^−1^. No clear change in the I_D_/I_G_ intensity ratio was observed for film thicknesses above 62.3 nm, whereas this ratio was smallest for the thinnest film (29.0 nm). The PLC film structure comprises three different layers: the surface, bulk, and interface layers. In addition, the surface and interface layers possess a lower density than the bulk layers for the same PLC films [20]. Furthermore, Harigai et al. reported that although the thickness of the bulk layer increased with the deposition time of CVD films, the thicknesses of the interface and surface layers were changed less in comparison [11]. However, the spectra determined using Raman spectroscopy or SE analysis are the average values for the entire structure of the PLC film; thus, it is difficult to differentiate between these layers. Therefore, samples with a lower PLC film thickness exhibited a decrease in the ratio of the bulk layer thickness to the total film thickness, suggesting that the Raman spectra and the optical constants are strongly influenced by the surface layer and substrate interface layer. Following the above explanation, the variation in the Raman spectra and optical constants as the film thickness decreases is considered to be a reasonable result. Figure 3 shows a structural image of the PLC film. The above results indicate that the classification of the bulk structural differences in PLC films based on optical constants requires the standardization of the film thickness.

### 3.3. Cell Proliferation Changes with Growth of the PLC Films

**Figure 4:**
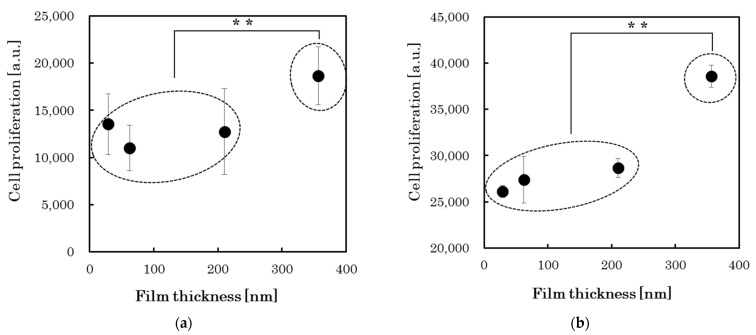
Cell proliferation of the PLC films with different thicknesses. (**a**) Fibroblasts (NIH-3T3); (**b**) osteoblast (MC-3T3). ** *p* < 0.01.

**Figure 5:**
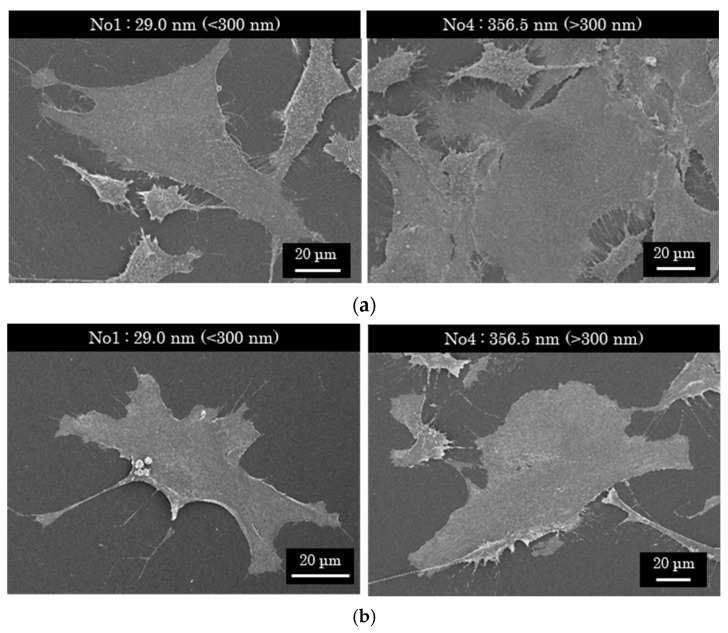
Cell morphology images of relatively thick (>300 nm) and thin (<300 nm) PLC films. (**a**) Fibroblasts (NIH-3T3); (**b**) osteoblast (MC-3T3).

In vitro cell culture testing was used to evaluate the cell proliferation properties of each PLC sample. Table 5 and Figure 4 present the cell proliferation for different PLC film thicknesses. During cell culture testing, the PLC films on the Si substrates were not peeled off. Instead, they were kept stable in the D-MEM or MEM-α solution. Thereafter, each cell was cultured for 72 h on the PLC films. The results demonstrate that fluorescent emissions, caused by the surviving cells equal to or higher than the control, were detected for all the PLC samples. It was found that all the PLC films prepared for this experiment were non-toxic toward the NIH-3T3 or MC-3T3 cells and showed favorable cytocompatibility. Furthermore, *p*-values were calculated to estimate the significant difference in cell proliferation for each film thickness. The results showed that there existed a statistically significant difference between samples with relatively thick films (>300 nm) and the other thin-film samples (*p* < 0.01), regardless of cell type. Figure 5 shows the cell morphology images of relatively thick (>300 nm) and thin (<300 nm) PLC films; the cell morphology on these films exhibited no clear difference between these films’ thicknesses and appeared to be stably attached. Although the effect of film thickness on each DLC type must be investigated in the future, it is possible that thicker PLC films could provide cell proliferation potential comparable to that of a-C or GLC types. This indicates that the changes in surface properties due to film growth must be considered when comparing the cell proliferation properties of DLC film types based on n–k plots. In addition, at least when PLC films are applied as a biological interface for medical devices, their growth (thickness), which affects the surface properties, needs to be carefully controlled.

### 3.4. Changes in Surface Properties with the Growth of PLC Films

The wettability, surface composition, and surface roughness of each PLC sample were evaluated using surface contact angle measurements with pure water, in addition to the XPS and AFM surface analyses. Table 5 shows the results of the surface analysis. The contact angle and surface roughness were expressed as the mean ± standard deviation. For the PLC films, the results of the contact angle with pure water, which were determined using the θ/2 method, indicated that thicker PLC films featured stronger hydrophilization than the thinner films. In a previous study, we confirmed that the wettability of a DLC surface affects the adhesion of NIH-3T3 cells [21]. This indicates that the hydrophilicity accompanying an increase in the thickness of the PLC films may have contributed toward the promotion of cell proliferation. Generally, wettability is dependent on the electrical and physicochemical properties of the PLC film surface.

**Figure 6:**
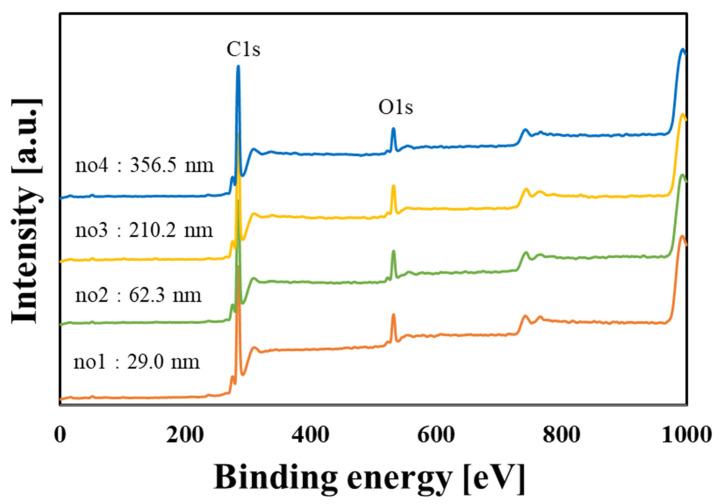
XPS wide scan spectra of the PLC films with different thicknesses.

**Figure 7:**
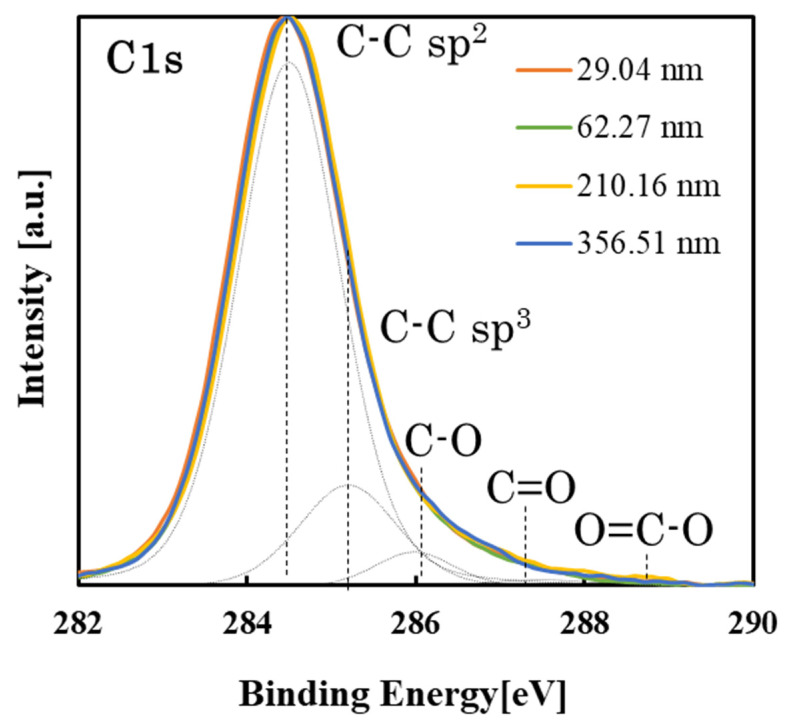
C1s spectra of the PLC films with different thicknesses. (The results of the no4 waveform separation spectrum are shown in the figure for reference).

XPS analysis was used to analyze the surface composition of the PLC films. Figure 6 and Figure 7 show the survey spectra and C1s spectrum, respectively. The survey spectra identified clear peaks for both C1s and O1s. However, the Si2p peak caused by the Si substrate was not detected. Therefore, it is suggested that the PLC film covers the Si substrate entirely in all the samples.

As shown in Table 5 and Figure 7, waveform separation of the C1s peaks of the PLC samples with different film thicknesses was resolved into C-C sp^2^, C-C sp^3^, C-O and C=O. Comparison of the peak intensity and C1s spectral shape of each of these components showed no significant differences in the surface composition of the four PLC samples. Accordingly, only small differences in the electrochemical action of the PLC samples prepared for this experiment were expected. In addition, the structure of DLC films affects the chemisorption state of the functional groups [22]. The lack of significant differences among the surface chemical compositions of the four PLC samples can be attributed to their similar bulk structures.

**Figure 8:**
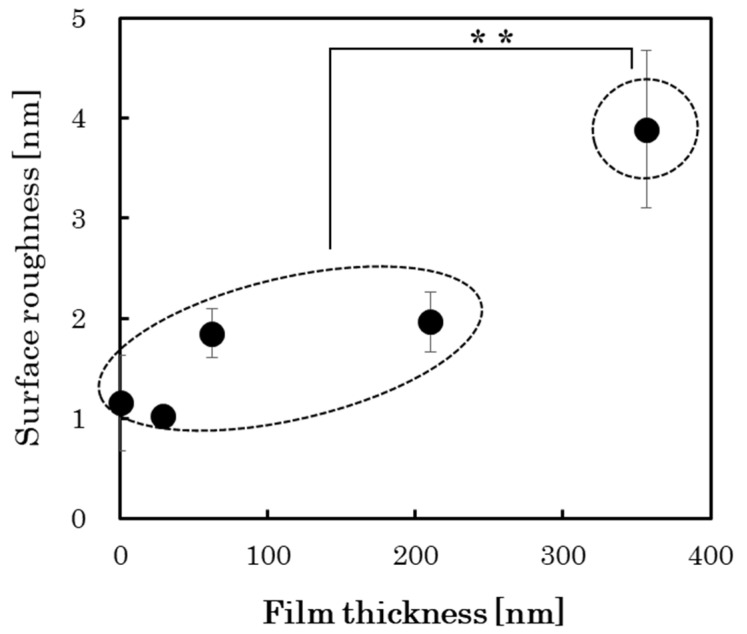
Surface roughness of the PLC films with different thicknesses. ** *p* < 0.01.

**Figure 9:**
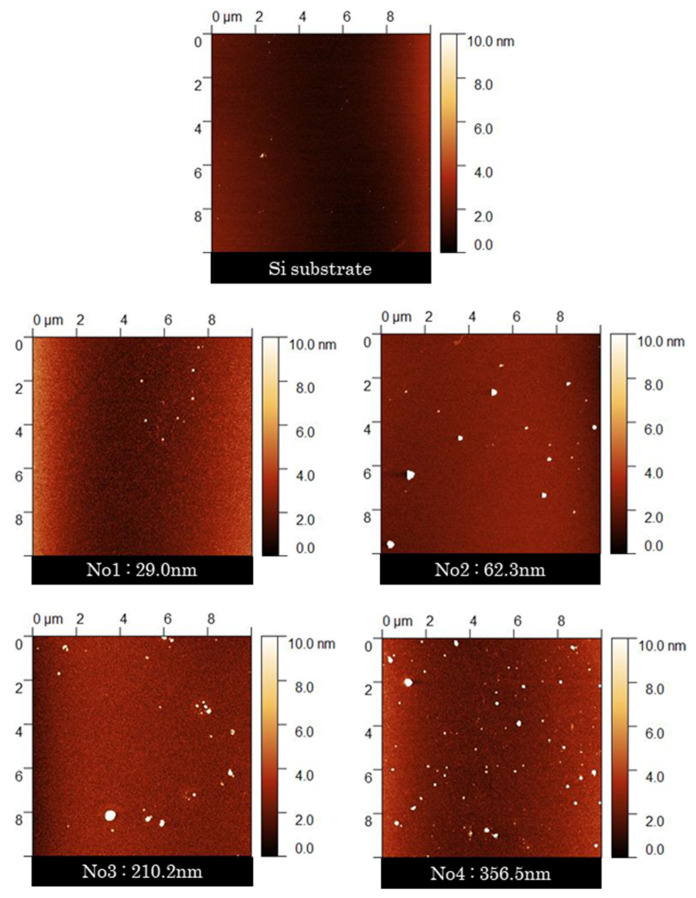
AFM images of the PLC samples.

AFM analysis was used to analyze the surface roughness of the PLC films with different thicknesses, as shown in Figure 8 and Figure 9. The resolution of the surface roughness was 0.01 nm. The results show that the surface roughness (RMS) tends to vary by several nanometers depending on the film growth of the PLC film. Furthermore, the difference between the surface roughness of the two groups divided according to PLC film thickness (Figure 4) was significant (*p* < 0.01), as confirmed by Student’s *t*-test. Accordingly, the increase in hydrophilicity with the PLC film growth can be attributed to the accompanying increase in surface roughness [23]. In addition, the structure of the material surface, which forms a foundation for the cells, has a significant influence on cell proliferation, shape changes, and cell differentiation [10]. The promotion of cell proliferation via the increased PLC film growth was thought to result from the corresponding changes in the physical surface roughness and wettability of the PLC film. The roughness of the DLC film is dependent on the surface roughness of the substrate [10]. This suggests that the selection of substrates for film deposition is also an important factor for PLC films that are standardized for biological responses.

## 4. Conclusions

This study investigated the correlation of cell proliferation with the surface properties of PLC films of different thicknesses prepared by radio-frequency plasma CVD. The thicknesses of these PLC films ranged from 29.0 to 356.5 nm, and all the samples were classified as PLC-type based on their n–k plots. The surface roughness and hydrophilicity, which are cell growth factors, then increased with the film growth (increase in film thickness) of the PLC films. As a result, samples with relatively thick PLC films (>300 nm) exhibited stronger cell proliferation properties than the thin-film samples.

These results indicate that when PLC films are selected for bio-interface applications, it is important to carefully control the film thickness, which affects their surface function.

## Figures and Tables

**Table 1:** Polymer-like carbon (PLC) film deposition conditions.

Sample No.	Deposition Method	Precursor	Power [W]	Gas Pressure [Pa]	Deposition Time [min]
1	13.56 MHz RF-PECVD	CH_4_ (37 sccm)	200	50	0.8
2					2.2
3					7.7
4					13.2

**Table 2:** Cell culturing conditions.

Cell	Fibroblast (NIH-3T3)	Osteoblast (MC-3T3)
Seeding density	1.0 × 10^4^ cells/cm^2^	1.0 × 10^4^ cells/cm^2^
Medium	D-MEM/F-12	MEM-α
CO_2_ concentration	5.0%	5.0%
Temperature	37.0 °C	37.0 °C
Incubation time	72 h	72 h
pH	6.8–7.2	6.8–7.2

**Table 3:** PLC film thickness and optical constants determined via spectroscopic ellipsometer analysis.

Sample No.	Film Thickness [nm]	n	k	χ^2^	Type
		λ = 550 nm			
1	29.0 ± 0.2	1.714	0.012	0.25	Polymer-like carbon
2	62.3 ± 0.4	1.763	0.014	0.87	
3	210.2 ± 1.1	1.803	0.030	2.35	
4	356.5 ± 3.4	1.830	0.031	5.52	

**Table 4:** Gaussian fitting results of Raman spectra.

Sample No.	D-Peak Position [cm^−1^]	G-Peak Position [cm^−1^]	I_D_/I_G_ Ratio
1	1337.06 ± 5.38	1515.77 ± 0.85	0.14
2	1328.68 ± 3.54	1521.66 ± 0.21	0.44
3	1336.72 ± 2.78	1523.71 ± 0.18	0.48
4	1334.38 ± 3.39	1524.85 ± 0.18	0.44

**Table 5:** Cell proliferation, wettability, surface composition, and surface roughness for each PLC sample.

Sample No.	Cell Proliferation (n = 3)		Compositional Bond Intensity of C1s Peaks					Pure Water Contact Angle [deg]	Surface Roughness RMS [nm]
	Fibroblast (NIH-3T3)	Osteoblast (MC-3T3)	C-Csp^2^	C-Csp^3^	C-O	C=O	O=C-O	Ave. (n = 10)	Ave. (n = 5)
1	13,546 ± 2930	26,116 ± 640	0.94	0.13	0.05	0.01	0.00	84.2 ± 0.8	1.03 ± 0.05
2	11,004 ± 2434	27,406 ± 2525	0.93	0.21	0.07	0.01	0.00	81.0 ± 0.7	1.85 ± 0.24
3	12,729 ± 4577	28,672 ± 1028	0.91	0.25	0.06	0.02	0.00	78.6 ± 0.8	1.96 ± 0.30
4	18,667 ± 3011	38,585 ± 1176	0.92	0.18	0.06	0.01	0.00	76.6 ± 0.8	3.89 ± 0.79
Si substrate	7233 ± 443	25,651 ± 2605	-	-	-	-	-	-	1.16 ± 0.48

## Data Availability

The data presented in this study are available on request from the corresponding author.
